# Comparison of Effects between Combined Lumbar-Sacral Plexus Block plus General Anesthesia and Unilateral Spinal Anesthesia in Elderly Patients Undergoing Hip Fracture Surgery: A Pilot Randomized Controlled Trial

**DOI:** 10.1155/2021/6685497

**Published:** 2021-04-30

**Authors:** Lili Tang, Panpan Fang, Yuxin Fang, Yao Lu, Guanghong Xu, Xuesheng Liu

**Affiliations:** Department of Anesthesiology, The First Affiliated Hospital of Anhui Medical University, Hefei 230022, China

## Abstract

**Purpose:**

Hip fracture is a common injury in geriatric populations, which is associated with poor quality of life. However, the ideal anesthesia technique for this disease is yet to be identified. This study aimed to compare the combined lumbar-sacral plexus block (CLSB) plus general anesthesia (bispectral index (BIS) 60–80) with the unilateral spinal anesthesia (SA) on activity of daily living in elderly patients undergoing hip fracture surgery.

**Methods:**

A total of 124 elderly patients undergoing hip fracture surgery were randomly assigned to two groups. Patients in the SA group received light-specific gravity spinal anesthesia, and patients in the CLSB group received lumbar and sacral plexus block with general anesthesia (BIS 60–80). The primary outcomes were 30-day activity of daily living (ADL). The secondary outcomes were postoperative pain scores, postoperative delirium, in-hospital cost, and major complications.

**Results:**

The ADL scores of postoperative day 30 (POD30) in the CLSB group are higher than those in the SA group (27.34 ± 7.01 versus 24.70 ± 6.40, *P*=0.045). Compared to preoperative ADL scores, there were higher increased scores in the CLSB group than in POD30 (CLSB group 8.09 ± 3.39 versus SA group 4.87 ± 3.90, *P* < 0.001). Mild-to-moderate pain did not have differences between the two groups (rest pain: 3 versus 2, *P*=0.344; motion pain: 5 versus 4, *P*=0.073). There were no significant differences in incidence of postoperative delirium, PONV, and other complications.

**Conclusion:**

The unilateral SA can reduce the deterioration of ADL after hip fracture surgery and provide a better postoperative recovery.

## 1. Introduction

Hip fracture is a common injury in geriatric populations, ranking the second among the causes of hospitalization and disability for elderly patients. It has critical consequences of deteriorated function status, for example, increasing mortality and decreasing quality of life [1, 2]. A large percentage of these patients did not attain their prefracture level of independence and ambulatory status [3, 4]. Thus, no matter for orthopedists or anesthesiologists, it is important to implement occupational therapy interventions to improve the function status and the quality of life among this population.

Activity of daily living (ADL), an important tool to assess the functional status and the quality of life, has been widely used to evaluate postoperative recovery and disease progression [5]. Previous studies had investigated the potential influence of different surgical methods [6], the early operative time [7], and the nutritional status [8] on the activities of daily living after hip fracture surgery. However, evidence to clarify the influence of different types of anesthesia on postoperative ADL is still lacking.

Both the combined lumbar and sacral plexus block (CLSB) with general anesthesia (BIS 60–80) and the unilateral spinal anesthesia (SA) on the operative side had been reported to be safe and effective as regional anesthesia techniques for hip surgery [9–11]. To the authors' knowledge, there were no studies comparing the superiority of these two anesthesia methods in terms of ADL. Hence, the purpose of this study was to compare the CLSB plus general anesthesia (BIS 60–80) and the unilateral spinal anesthesia plus monitored anesthesia care (MAC) on the postoperative ADL of elderly patients undergoing hip fracture surgery. We hypothesized that the unilateral SA plus MAC would attenuate the deterioration of daily activity compared to the CLSB plus general anesthesia.

## 2. Materials and Methods

This prospective, controlled, and two parallel-group clinical trial was approved by the ethics committee of the First Affiliated Hospital of Anhui Medical University (approval number: PJ2018-11-06) and registered in the Chinese Clinical Trial Registry (ChiCTR1900025113; principal investigator: Panpan Fang; date of registration: August 12, 2019). The study was conducted in accordance with the principles of the Helsinki Declaration at the First Affiliated Hospital of Anhui Medical University.

### 2.1. Patients and Randomization

Between August 2019 and December 2019, patients who underwent elective unilateral hip fracture surgeries, including osteosynthesis, artificial femoral head replacement, and total hip replacement, at the First Affiliated Hospital of Anhui Medical University were assessed for eligibility.

The inclusion criteria were as follows: >65 years of age and American Society of Anesthesiologists I–IV. The exclusion criteria were as follows: dementia or severe cognitive dysfunction (simple mental state questionnaire ≥8), unstable mental state or mental disease, reception of psychotropic drugs or abuse of narcotic sedation analgesics, being delirious or history of delirium, anesthesia and surgery within 6 months, other surgeries at the same time, cerebrovascular accidents such as cerebral stroke and transient ischemic attack within 3 months, and prosthesis fracture repair surgery.

The participants were randomized to the CLSB or the SA group with a 1 : 1 allocation using computer-generated randomized numbers. Patients in the CLSB group received lumbar and sacral plexus block with general anesthesia (BIS 60–80), while patients in the SA group received light-specific gravity spinal anesthesia plus MAC. Written informed consent forms were offered to all patients or their legal relatives.

### 2.2. Anesthetic Management

Heart rate, blood pressure, electrocardiograph, pulse oxygen saturation (SpO_2_), and BIS were routinely monitored for each patient. After the venous access was available, all patients were injected with 6–8 mL/kg/h of Ringer's lactate solution or hydroxyethyl starch 130/0.4 sodium chloride before anesthesia. Then, the fluids were adjusted according to the hemodynamic monitoring and blood loss in the operating room.

### 2.3. CLSB with General Anesthesia (BIS 60–80)

All CLSB procedures were conducted by an attending anesthesiologist well versed with peripheral anesthesia. Before the patient was placed in a lateral decubitus position with the operated side uppermost, flexion of uninjured hip and knee, 5 *μ*g sufentanil was administered intravenously for pain relief.

During the CLSB procedure, oxygen was provided via a face mask with a flow of 5–8 L/min. All peripheral nerve blockades were performed under the guidance of a nerve stimulator (Stimuplex HNS 12, B. Braun Medical Inc., Germany) and ultrasound (FUJIFILM Sonosite Inc., WA, USA). A 12 cm 22-gauge nerve stimulation needle (Stimuplex D, B. Braun Medical Inc., Germany) was advanced perpendicularly to the skin between L3 and L4 transverse processes. An appropriate needle position was confirmed as quadriceps contraction after a stimulating current of 0.4 mA and a frequency of 2 Hz. After negative aspiration with blood, 20 mL of 0.25% ropivacaine was slowly injected.

The sacral plexus nerve block was performed using the transgluteal approach with the same position under ultrasound and nerve stimulation guidance. The sacral plexus was identified by the motor response of the gluteus maximus and gastrocnemius, with a stimulating current of 0.4 mA and a frequency of 2 Hz. Similarly, 20 mL of 0.25% ropivacaine was slowly injected to complete the sacral plexus block. It was considered as a failed procedure in the case of the absence of the right motor responses after three nerve block puncture attempts. Then general anesthesia was applied, and the patient was eliminated from the study.

After confirming successful block using the pinprick test, propofol (1–1.5 mg/kg), sufentanil (0.1–0.2 *µ*g/kg), and cisatracurium besilate (0.2 mg/kg) were used for anesthesia induction [10]. A laryngeal mask (LMA Supreme, Laryngeal Mask Company Ltd., Malaysia) was used for airway management. Mechanical ventilation was set as follows: the tidal volume was set as 6–8 mL/kg, the respiratory rate was set as 10–12 breaths/min, the ratio of expiration: inspiration was set as 2 : 1, and the end-tidal carbon dioxide pressure remained at 35–40 mmHg. The effect-site concentration of propofol was adjusted to maintain the depth of sedation (BIS: 60–80) [10]. The depth of sedation was assessed by observer's assessment of alertness/sedation (OAA/S) as a supplement.

### 2.4. Unilateral Spinal Anesthesia plus MAC: Light-Specific Gravity Spinal Anesthesia

All the SA procedures were performed by a skilled attending anesthesiologist. After the patient was placed in a vertical position with the operated side uppermost and flexion of uninjured hip and knee, the puncture sites were selected at L3–4 or L2–3 vertebral interspace. The median side of the spine was opened between 0.5 and 1.0 cm for local infiltration. With both hands holding the protruding needle tip (25 G), the needle was advanced perpendicularly into the spine through the middle of the lumbar vertebral space. The recorded depth was between 3.5 and 5.0 cm. Once encountering bone, the needle tip was slightly tilted to the side of the head by about 10° to 15° and then reinserted until the clear cerebrospinal fluid reflux. Further, 4 mL of 0.25% hypobaric ropivacaine was injected into the subarachnoid space [12]. The sensory block on lower limbs was evaluated using the pinprick test, whereas the motor blockade was evaluated by modified Bromage scale (0 = no motor block, 1 = hip blocked, 2 = hip and knee blocked, and 3 = hip, knee, and ankle blocked). Assessments of motor and sensory blocks in the operated and the nonoperated sides were made at the following times: 3, 5, 10, and 15 minutes after the injection. Successful anesthesia was defined as no pain at T12 and a modified Bromage score ≥2 only on the operated limb. Then, light depth of sedation (BIS 60–80) was maintained by adjusting the effect-site concentration of propofol. The depth of sedation was assessed by observer's assessment of alertness/sedation (OAA/S) as a supplement. The absence of the right motor response after three spinal puncture attempts was considered as failed, and then general anesthesia was applied.

Intraoperative hypotension was defined as a decrement in systolic blood pressure by more than 20% from preoperative values and/or mean arterial pressure less than 65 mmHg. Patients with intraoperative hypotension were immediately treated with phenylephrine. All operations were completed using the posterior approach and by the same arthroplasty surgeons. All patients were transferred to the postanesthesia care unit (PACU) after showing satisfactory spontaneous breathing.

The criteria for extubation were as follows: recovery of consciousness, the train-of-four ratio >0.9, the tidal volume >6 mL/kg, the breathing rate <30 beats/min, and the maintenance of pulse oximetry (SpO_2_) at >92% under air inspiration. Postoperative analgesia was achieved by a patient-controlled analgesia (PCA) solution, which was prepared by dissolving sufentanil (2.5 *μ*g/kg) and flurbiprofen axetil (100 mg) in 100 ml of saline. For the loading dose, one bolus was set to 2 mL and background infusion rate was set to 2 ml/h, while the lockout time was set to 15 min. The analgesic goal was postoperative VAS ≤3. If the VAS score ≥4, patients would receive rescue analgesic to relief pain.

### 2.5. Outcomes Measures

#### 2.5.1. Primary Endpoint

The primary endpoint was the 30-day function status assessed by the Chinese version of the daily living scale (CADL), which includes a physical self-maintenance scale (PSMS) and an instrumental activities of daily living (IADL) scale [13]. The function status before fracture was assessed one day before surgery with CADL by interviewing the patients. The PSMS, normally used to assess the fundamental skills to live, consists of six tasks: ambulating, dressing, eating, grooming, toileting, and bathing. The IADL scale is used to assess more complicated activities that allow an individual to live independently. This scale contains eight tasks: preparing food, taking public transportation, housekeeping, doing laundry, taking medications, making phone calls, shopping, and managing finances. CADL has a total of 14 items and ranged from 14 to 56 points. The single item score is determined by a 4-point numeric rating scale (1 = can do it by oneself, 2 = have some difficulty but can still do it by oneself, 3 = need help to do it, and 4 = cannot do it at all). Higher ADL scores indicate a poor functional status, and 22 points were the cutoff score. Over the cutoff score was defined as impairment in ADL.

#### 2.5.2. Secondary Endpoints

Secondary endpoints included the incidence of postoperative delirium, pain scores, the in-hospital cost, postoperative nausea and vomiting (PONV), and major postoperative complications (stroke and heart failure). All the patients had same discharge criteria: no fever, resumed a normal diet, and well incisions heal. The preoperative mental state was assessed using the simple mental state questionnaire, while perioperative delirium was evaluated by the Confusion Assessment Method (CAM) [14]. The postoperative delirium was assessed twice daily for 7 days or until discharged. Rest pain, motion pain, postoperative nausea and vomiting (PONV), and major postoperative complications were evaluated at 8 : 00 a.m. on the first three days after surgery. Evaluation for ADL score was completed via phone calls on the 30th day after surgery (postoperative day 30, POD30).

A trained researcher in our department conducted all these assessments, and he was blinded to the randomized allocation and intervention in this study.

### 2.6. Statistical Analysis

We used PASS11.0 (NCSS, LLC., Kaysville, UT, USA) for the sample size calculation. According to our pilot data, we estimated that the mean of ADL incremental value was 5 in SA group and the SD was 4; the mean of ADL incremental value was 8 in CLSB group. Assuming a 5% two-tailed type I error rate, a sample size of 92 was required to detect a significant difference with the power of 90%. Allowing for 15% noncompliance, at least 106 patients were required in the present study.

Data were expressed as mean ± standard deviation, median (interquartile range), or number (proportion, %) and analyzed by SPSS version 16.0 (SPSS Inc., IL, USA). For normal-distribution data, two-tailed Student's *t*-test was used. Continuous data that were not normally distributed were analyzed by the Mann–Whitney *U* test. Categorical data were analyzed using the chi-square (*χ*^2^) test or Fisher's exact test where appropriate. Statistical significance was set at *P* < 0.05.

## 3. Results

**Figure 1:**
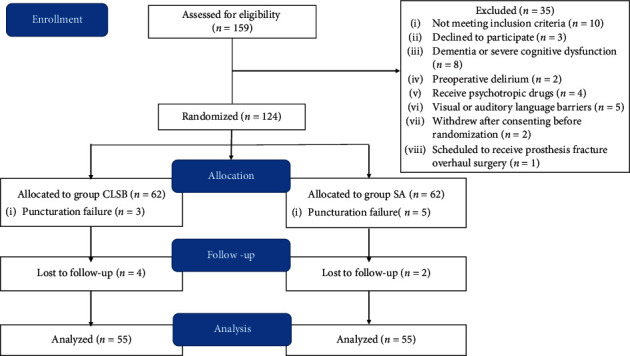
The consort flow chart outlining patients' assignment and treatment protocols. Patients in the SA group received light-specific gravity spinal anesthesia, while the CLSB group received lumbar and sacral plexus block with light sedation (BIS 60–80).

Of the 159 patients assessed for eligibility, 10 patients did not meet the inclusion criteria, while 3 refused to participate in the study. Further, 22 patients were excluded from the study. Eventually, 124 patients were randomly assigned to SA and CLSB groups. In the CLSB group, four patients were lost to follow-up and three patients were switched to general anesthesia. In the SA group, two patients were lost to follow-up and five were switched to general anesthesia. Therefore, only 110 patients completed the study (Figure 1). No significant differences were observed in the demographics and baseline data between two groups (shown in Table 1).

### 3.1. Primary Outcomes

**Figure 2:**
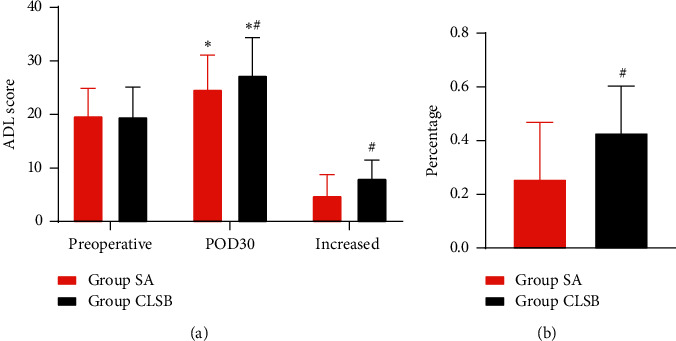
ADL score, percentage of increased ADL scores between the two groups. (a) ADL scores of two groups. (b) Percentage of two groups. ^*∗*^*P* < 0.05, compared with preoperative ADL scores; ^#^*P* < 0.05, the CLSB group compared with the SA group; ADL, activity of daily living; CLSB, combined lumbar and sacral plexus block; POD, postoperative day; SA, spinal anesthesia. A higher ADL score means a worse quality of life.

The ADL scores before fracture were comparable between two groups. The patients in the CLSB group had a higher POD30 ADL score (27.34 ± 7.01 versus 24.70 ± 6.4, *P*=0.045) (shown in Table 2). The ADL scores of POD30 in both groups were increased, compared with the ADL scores before fracture. However, patients in the CLSB group had higher incremental ADL values (8.09 ± 3.39 versus 4.87 ± 3.90, *P* < 0.001) and a higher decremental function level (43.04% versus 25.83%, *P* < 0.001) compared with the patients in the SA group (Figure 2).

### 3.2. Secondary Outcomes

In addition, patients in the SA group had a lower in-hospital cost (44264 ± 9115.12 versus 49636 ± 9708.11, *P* < 0.003) compared with ones in the CLSB group. There was no patient having stroke or heart failure in both groups. No significant difference was found in the incidence of PONV, postoperative delirium, blood transfusion, and the postoperative pain score between the two groups (shown in Table 2).

No significant difference was observed between two groups in terms of intraoperative crystal, colloid infusion, and intraoperative blood loss. Compared with the CLSB group, patients in the SA group had a shorter residence time in PACU (51.27 ± 14.51 versus 39.82 ± 10.38, *P* < 0.001). Time of surgery, time of anesthesia, and the incidence of hypotension were comparable between two groups. The type of surgery was comparable between the two groups (Table 3).

## 4. Discussion

The results of this study showed a fewer loss of the daily activity in the SA group, compared with the patients in the CLSB group, although the patients in both groups did not return to the preoperative functional status 30 days after surgery. Furthermore, the rate of ADL decrement in the CLSB group was higher than that in the SA group. More items' scores were 3 (need help to do it) to 4 (cannot do it at all) in the CLSB group. Moreover, the patients in the SA group exhibited a shorter residence time in PACU and a lower in-hospital cost.

The functional status and the quality of life after hip fracture surgery are common concerns for orthopedics and anesthesiologists. It has been reported that nearly 33.3% of patients failed to return their prefracture ADL at six months after hip surgery [3]. Kristensen [4] reported that 13% older patients with hip fracture are no longer able to live alone. In line with previous studies, our data demonstrated that POD30 ADL did not return to the preoperative state through aggressive and proper surgical treatments.

In the current study, the items in PSMS are necessary for basic functional living and the ability to implement IADLs can significantly improve the quality of life. A higher CADL score (impaired ADL) may indicate the need for home healthcare or more medical resources [15]. Our study demonstrated that the CADL score in the CLSB group was also higher than that in the SA group at POD30. This indicates that the patients in the CLSB group perceived greater physical functional disability. Segev-Jacubovski et al. reported that the improved functional ability achieved by therapy intervention can promote health-related quality of life among elderly with hip fracture [16]. Hence, patients in the CLSB group with a higher rate of declined ADL ability need more therapy intervention to recovery.

No difference in the postoperative pain score was found between groups. This may be associated with the successful block of the both groups and the effective postoperative controlled analgesia. Small dose of sufentanil with propofol (1–1.5 mg/kg) in CLSB was just to reduce or avoid the effect of LMA insertion. This method is consistent with the previous study [10]. It is also consistent with previous findings [17, 18] that patients treated with spinal anesthesia had a shorter stay time in PACU and a lower in-hospital cost. Spinal anesthesia was not widely applied in patients undergoing hip surgery due to the potential hemodynamic compromise and urinary retention. However, the superiority of the unilateral spinal anesthesia was confirmed in outpatients' surgery with fewer complications [19]. Unilateral SA reduced the consumption of general anesthetics and avoided the artificial airway with an exact effect of nerve blocking.

Postoperative delirium is a common complication in elderly patients undergoing hip fracture surgery [20]. However, in the current study, the incidence of postoperative delirium was 5.5% in the SA group and 7.3% in the CLSB group. The lower prevalence of delirium may partially be attributed to strict exclusion of participants and decreased opioids after application of regional anesthesia [10].

The present study had some limitations. Firstly, the number of PCA boluses and the time of the first postoperative requirement of analgesics were not recorded. Secondly, it is limited to assess the chronic pain 30 days after surgery in our study. Thirdly, the follow-up was only to 30 days after surgery. Therefore, the results of long-term ADL need to be investigated in our future study.

In conclusion, the results indicated that the unilateral SA plus MAC can reduce the deterioration of ADL after hip fracture surgery and can provide a better postoperative recovery. In addition, less cost was found in the SA group. Hence, the unilateral SA plus MAC may be more suitable than CLSB for elderly patients undergoing hip fracture surgery.

## Figures and Tables

**Table 1:** Demographics and baseline data of the study population.

	Group SA	Group CLSB	*P* value
Age (year)	78.00 (6.45)	76.60 (6.98)	0.277
Sex (male/female)	16/39	20/35	0.416
BMI	21.35 (3.16)	22.41 (3.25)	0.086
ASA (II/III/IV)	21/31/3	21/29/5	0.818
*CCI*			0.205
0	14/55 (25.5%)	11/55 (20.0%)	
1	17/55 (30.9%)	13/55 (23.6%)	
2	20/55 (36.4%)	25/55 (45.5%)	
3	3/55 (5.5%)	4/55 (7.3%)	
4	1/55 (1.8%)	2/55 (3.6%)	
*Education (year)*			0.079
0	28/55 (50.9%)	19/55 (34.5%)	
5	13/55 (23.6%)	18/55 (32.7%)	
8	6/55 (10.9%)	4/55 (7.3%)	
11	7/55 (12.7%)	9/55 (16.4%)	
15	1/55 (1.8%)	4/55 (7.3%)	
18	0/55 (0.0%)	1/55 (1.8%)	
Hemoglobin (g/dL)	10.99 (1.87)	11.34 (2.34)	0.397
Albumin (g/dL)	3.74 (0.41)	3.78 (0.55)	0.619
Preoperative ADL	19.76 (5.11)	19.60 (5.54)	0.872
Rest pain	3 (2, 4)	2 (2, 3)	0.344
Motion pain	5 (4, 6)	4 (3, 6)	0.073

Statistics are presented as mean (standard deviation), median (interquartile range), or *N*/total number of patients (%) as appropriate. ADL, activity of daily living; ASA, American Society of Anesthesiologists; BMI, body mass index; CCI, Charlson comorbidity index; CLSB, combined lumbar and sacral plexus block; SA, spinal anesthesia.

**Table 2:** Postoperative outcomes.

	Group SA	Group CLSB	*P* value
POD30 ADL	24.70 (6.40)	27.34 (7.01)	0.045 *∗*
ADL increased value	4.87 (3.90)	8.09 (3.39)	<0.001 *∗*
POD1 rest pain	0 (0, 2)	1 (0, 2)	0.131
POD1 motion pain	2 (1, 4)	3 (2, 4)	0.208
POD2 rest pain	0 (0, 2)	1 (0, 2)	0.132
POD2 motion pain	2 (1, 3)	3 (2, 3)	0.058
POD3 rest pain	0 (0, 1)	0 (0, 1)	0.068
POD3 motion pain	2 (1, 2)	2 (1, 2)	0.143
Postoperative delirium	6/55 (5.5%)	8/55 (7.3%)	0.567
POD1 PNOV			0.152
1^a^	47/55 (85.5%)	41/55 (74.5%)	
2^a^	6/55 (10.9%)	10/55 (18.2%)	
3^a^	2/55 (3.6%)	4/55 (7.3%)	
POD2 PONV			0.243
1^a^	53/55 (96.4%)	50/55 (90.0%)	
2^a^	2/55 (3.6%)	5/55 (9.1%)	
3^a^	0/55 (0.0%)	0/55 (0.0%)	
Blood transfusion	6/55 (10.9%)	9/55 (16.4%)	0.405
In-hospital cost (RMB)	44264 (9115.12)	49636 (9708.11)	0.003*∗*

Statistics are presented as mean (standard deviation), median (interquartile range), or *N*/total number of patients (%) as appropriate. ^*∗*^*P* < 0.05, CLSB group compared with SA group. ADL, activity of daily living; CLSB, combined lumbar and sacral plexus block; POD, postoperative day; PONV, postoperative nausea and vomiting; SA, spinal anesthesia. Grade of PONV: 1^a^, no nausea or vomiting; 2^a^, mild nausea and vomiting without no vomitus; 3^a^, severe nausea and vomiting with vomited matter.

**Table 3:** Intraoperative information.

	Group SA	Group CLSB	*P* value
Time of surgery (min)	70.42 (21.77)	71.35 (19.85)	0.816
Time of anesthesia (min)	78.62 (22.99)	76.49 (20.45)	0.609
PACU standing time (min)	39.82 (10.38)	51.27 (14.51)	<0.001 *∗*
Crystalloids (mL)	600 (600, 1000)	600 (600,1100)	0.236
Colloids (mL)	500 (0,500)	500 (0,500)	0.792
Blood loss (mL)	100 (100,200)	150 (100,200)	0.652
*Type of surgery*			0.244
Osteosynthesis	25/55 (45.5%)	19/55 (34.5%)	
Artificial femoral head replacement	16/55 (29.1%)	18/55 (32.7%)	
Total hip replacement	14/55 (25.5%)	18/55 (32.7%)	
Incidence of hypotension	15/55 (27.3%)	10/55 (18.2%)	0.257

Statistics are presented as mean (standard deviation), median (interquartile range), or *N*/total number of patients (%) as appropriate. ^*∗*^*P* < 0.05, CLSB group compared with the SA group. CLSB, combined lumbar and sacral plexus block; PACU, postanesthesia care unit; SA, spinal anesthesia.

## Data Availability

The data used to support the findings of this study are included within the article.
